# Virus-Induced Gene Silencing as a Tool for Comparative Functional Studies in *Thalictrum*

**DOI:** 10.1371/journal.pone.0012064

**Published:** 2010-08-10

**Authors:** Verónica S. Di Stilio, Rachana A. Kumar, Alessandra M. Oddone, Theadora R. Tolkin, Patricia Salles, Kacie McCarty

**Affiliations:** Department of Biology, University of Washington, Seattle, Washington, United States of America; University of Melbourne, Australia

## Abstract

Perennial woodland herbs in the genus *Thalictrum* exhibit high diversity of floral morphology, including four breeding and two pollination systems. Their phylogenetic position, in the early-diverging eudicots, makes them especially suitable for exploring the evolution of floral traits and the fate of gene paralogs that may have shaped the radiation of the eudicots. A current limitation in evolution of plant development studies is the lack of genetic tools for conducting functional assays in key taxa spanning the angiosperm phylogeny. We first show that virus-induced gene silencing (VIGS) of a *PHYTOENE DESATURASE* ortholog (*TdPDS*) can be achieved in *Thalictrum dioicum* with an efficiency of 42% and a survival rate of 97%, using tobacco rattle virus (TRV) vectors. The photobleached leaf phenotype of silenced plants significantly correlates with the down-regulation of endogenous *TdPDS* (P<0.05), as compared to controls. Floral silencing of *PDS* was achieved in the faster flowering spring ephemeral *T. thalictroides*. In its close relative, *T. clavatum*, silencing of the floral MADS box gene *AGAMOUS* (*AG*) resulted in strong homeotic conversions of floral organs. In conclusion, we set forth our optimized protocol for VIGS by vacuum-infiltration of *Thalictrum* seedlings or dormant tubers as a reference for the research community. The three species reported here span the range of floral morphologies and pollination syndromes present in *Thalictrum*. The evidence presented on floral silencing of orthologs of the marker gene *PDS* and the floral homeotic gene *AG* will enable a comparative approach to the study of the evolution of flower development in this group.

## Introduction

*Thalictrum*, in the buttercup family Ranunculaceae, comprises approximately 190 species globally distributed in temperate regions [1]. The genus exhibits a range of floral morphologies including four breeding systems and two pollination syndromes [2]. Commonly known as “meadow rues”, these perennial woodland herbs have been actively studied for the medicinal value of their secondary metabolites [3], [4], [5]. This lineage is ideally suited for the study of the origins of core eudicot diversity because of: (1) Its basal phylogenetic position within the eudicots and (2) The presence of ancestral floral traits, such as free, uniovulate carpels with ascidiate (open) development and variable number of spirally arranged floral organs [6].

A major hurdle in obtaining functional data from emerging model systems like *Thalictrum*, is a lack of transgenic techniques and genomic tools that are readily available for established model plants such as *Arabidopsis thaliana*. A single report of stable transgenesis in *Thalictrum* involves cell culture, with a low efficiency of explant regeneration [7]. The advent of virus-induced gene silencing (VIGS) by tobacco rattle virus (TRV) as a laboratory technique [8], offered a fast and effective solution to the need for functional data, and promises to bridge the gap between established and emerging model plant systems [9], [10].

VIGS was developed as a way of harnessing the RNA-mediated post-transcriptional gene silencing (PTGS) defense system naturally present in plants and other organisms to fight pathogens (reviewed in [11], [12], [13]). The technique relies on the use of viral vectors carrying a transgene that can trigger the PTGS system, causing the degradation of its homolog within the plant. One such viral vector is based on TRV and consists of a binary transformation system, pTRV1 and pTRV2, the latter carrying one or more transgene/s. TRV has been the virus of choice in a variety of plant species due to its minimal pathogenic effects, its wide host range and its ability to cause infection to meristematic tissues, including flowers [8].

Initially developed in members of the Solanaceae [14], [15], [16], [17], [18], VIGS has proved useful in several other plants species. For example, in *Petunia* it has been used to help elucidate mechanisms of floral scent production [19], while in soybean it has facilitated the dissection of the flavonoid biosynthetic pathway [20]. The application of such a convenient, fast and cost-effective tool is facilitating more comprehensive comparisons of gene function across diverse plant taxa, including monocots and basal eudicots [21], [22], [23], [24], [25], [26], [27].

*PHYTOENE DESATURASE* (*PDS*) encodes an enzyme that catalyzes an important step in the carotenoid biosynthesis pathway [28]. Silencing of this enzyme blocks the production of carotenoids (umbrella pigments for chlorophyll), causing the photodegradation of chlorophyll and consequently giving plants an easily recognizable photobleached appearance.

Our goal was to generate loss-of function phenotypes in the early-diverging eudicot *Thalictrum*, in order to understand gene function and enable a comparative approach. To that end, we first show the successful implementation of VIGS in seedlings of *T. dioicum*, by silencing the ortholog of the *PDS* marker gene, *TdPDS*, in leaves. Subsequently we apply a modified protocol to tubers of two fast-flowering spring ephemeral species and show silencing of *PDS* and an *AG* ortholog in flowers. These three species span the range of floral morphologies present in *Thalictrum*: wind pollinated, inconspicuous flowers with green sepals (*T. dioicum*) and showy, insect pollinated flowers with petaloid sepals (*T. thalictroides*) or petaloid stamens (*T. clavatum*) [29].

This approach will be subsequently applied to unravel the functional significance of other genes in these and related species. For example, it will allow to extend the study of previously described gene duplications undergone by critical flower transcription factors, such as the B and C class MADS box genes, to this early-diverging eudicot [30], [31].

## Results

### Silencing of *PDS* in leaves of *T. dioicum*

Our initial goal was to test whether the VIGS approach would be successful in our study system. To that end we set out to silence the ortholog of *PHYTOENE DESATURASE*, commonly used as a marker due to the easy-to-score resulting photobleached phenotype.

**Figure 1:**
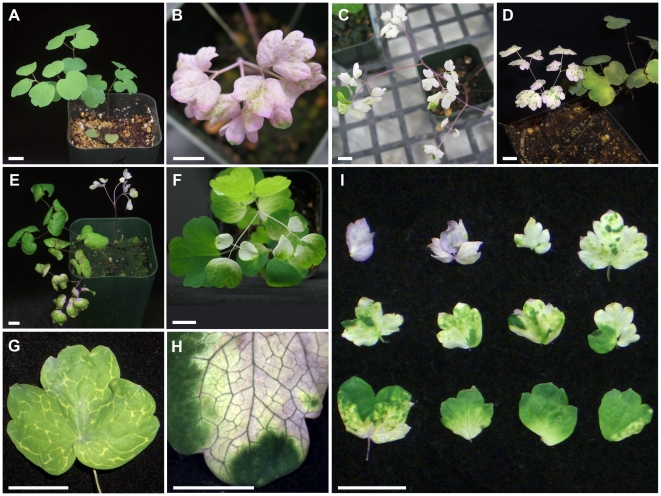
VIGS of *Thalictrum dioicum PHYTOENE DESATURASE* ortholog *TdPDS* results in varying degrees of leaf photobleaching. A: Untreated *T. dioicum* plant. B–F: Distribution of photobleaching in TRV2-*TdPDS* treated plants. G: Leaflet showing signs of silencing along the vascular tissue. H: Detail of partially photobleached leaflet. I: Typical range of silencing in TRV2-*TdPDS* treated leaflets. Scale bar  = 1 cm.

The overall survival rate of treated and mock-treated plants was 97%, indicating that *Thalictrum dioicum* seedlings are hardy and resilient to vacuum infiltration. Initiation of photobleaching in TRV2-*TdPDS* treated plants was observed approximately 2 weeks post-infiltration; after 2 months 42% of treated plants showed some degree of *TdPDS* silencing. Twelve percent of treated plants showed strong silencing, where a whole compound leaf, including the petiole, was photobleached, as compared to untreated plants (compare Fig. 1A to B–E). Intermediate phenotypes included scattered sectors of white throughout the plant (Fig. 1F), and milder ones exhibited photobleaching restricted to the vasculature of leaflets (Fig. 1G). Photobleached leaves often looked pink, due to the natural presence of anthocyanins, which were exposed by the photo-degradation of chlorophyll (Fig. 1B, H and I, first two leaflets). Overall, there was a gradient of silencing phenotypes at the leaflet level (Fig. 1I). The duration of silencing varied from six to eight weeks from onset, with a few outliers in which silencing continued for up to three months. Photobleached tissue was more vulnerable and typically died off over time, causing an overall apparent decline of silencing over time. Mock-treated plants were undistinguishable from untreated plants (not shown), suggesting no visible viral effects in this species at the vegetative level.

**Figure 2:**
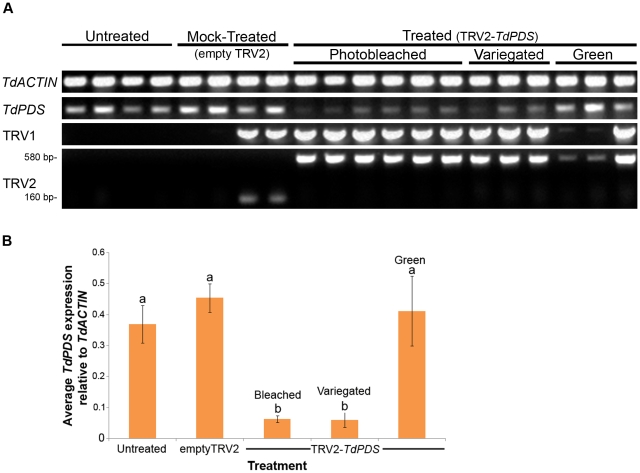
Downregulation of *TdPDS* and detection of TRV transcripts in VIGS photobleached leaves of *Thalictrum dioicum*. **A: Expression of** ***TdACTIN*** **control, native** ***TdPDS*** **and viral transcripts in leaves by Reverse Transcriptase (RT)-PCR.** Untreated and mock-treated (empty TRV2) *T. dioicum* plants are compared to TRV2-*TdPDS* treated plants showing photobleached (white), variegated (green/white) and green leaf tissue. RT-PCR was performed with locus-specific primers to the housekeeping gene *ACTIN* (loading control), to endogenous *TdPDS* and to the viral transcripts TRV1/TRV2. Approximate band size indicated for TRV2: larger band results from the presence of the *TdPDS* insert, smaller band from an empty TRV2 (mock control). **B: Comparative expression of** ***TdPDS*** **normalized with** ***TdACTIN*** **among treatments and resulting phenotypes of** ***Thalictrum dioicum*** **.** Values based on quantification of RT-PCR gel bands in part A using ImageJ (see text for details). Different letters indicate statistical significance in a one-way ANOVA followed by Tuckey test (p<0.05), same letters indicate no statistical difference. Average and standard error bars are shown. Sample sizes are n = 4 for untreated and mock-treated, n = 6 for treated bleached and n = 3 for treated variegated or green.

In order to confirm that the leaf photobleached phenotypes described above correlated with reduced endogenous levels of *TdPDS*, we performed Reverse Transcriptase (RT) PCR with locus-specific primers on leaf tissues from each of the three treatment groups (Fig. 2). Amplification of the *ACTIN* ortholog, *TdACTIN* was used as a template concentration control (Fig. 2A, top gel). To test if the phenotype observed in treated plants was due to the presence of the viral vectors, the presence of TRV1 and TRV2 transcripts in cDNA was also determined by RT-PCR (Fig. 2A, bottom 2 gels). Samples from the untreated group did not show viral expression and had high expression of *TdPDS*, as expected. Half of the mock-treated plants shown in Fig. 2 had both vectors, consistent with the 42% observed incidence of photobleaching in the TRV2-*TdPDS* treatment. RT-PCR performed with TRV2-specific primers spanning the multiple cloning site produced a smaller product size (160 bp) in two of the mock-treated plants, corresponding to the distance between primers in the absence of insert, therefore confirming the presence of TRV2 and the absence of the *TdPDS* transgene fragment (Fig. 2A, smaller bands in TRV2 panel). The same two plants also amplified TRV1 transcript. Expression of *TdPDS* in this treatment group was similar to that of untreated plants, suggesting that the viral treatment does not interfere with *TdPDS* expression. We further subdivided the pTRV2-*TdPDS* treatment into three categories based on silencing phenotype intensity: green (from partially silenced plants), variegated (green leaflets with white silenced sectors) and completely photobleached tissues (white leaflets). All of the TRV2-*TdPDS* treated photobleached plants showed presence of transcript from both vectors. Detection of the *TdPDS* transgene in pTRV2 is indicated by the larger PCR product size (Fig. 2A, 585 bp band in TRV2 panel).

Quantification of band intensity (from the RT-PCR gels in Fig. 2A) confirmed a statistically significant down-regulation of *TdPDS* (relative to *ACTIN*) in fully photobleached and variegated leaf samples compared to untreated and mock-treated controls and treated-green leaves (p<0.05, denoted by different letters on top of the bars in Fig. 2B). The decrease in levels of endogenous *TdPDS* in bleached and variegated leaves was not statistically significant at the resolution allowed by RT-PCR (equal letters above bars in Fig. 2B), a more quantitative expression method may be needed to detect these more subtle differences. For our purposes, variegated leaves may be considered as silenced. Green leaves from plants that had shown silencing in other leaf tissue had endogenous *TdPDS* levels undistinguishable from the untreated or mock-treated plants, indicating that treated plants are chimeras of silenced and non-silenced tissue for *TdPDS*.

Since silencing lasts for 2–3 months, it became apparent that the time to flowering in seedlings of *T. dioicum* is typically greater (4–6 months) than the duration of our silencing phenotypes. To implement VIGS to the study of flower development we extended the silencing assays to include faster flowering species within the genus.

### Floral silencing in fast-flowering spring ephemerals

#### PDS silencing in *T. thalictroides*

**Figure 3:**
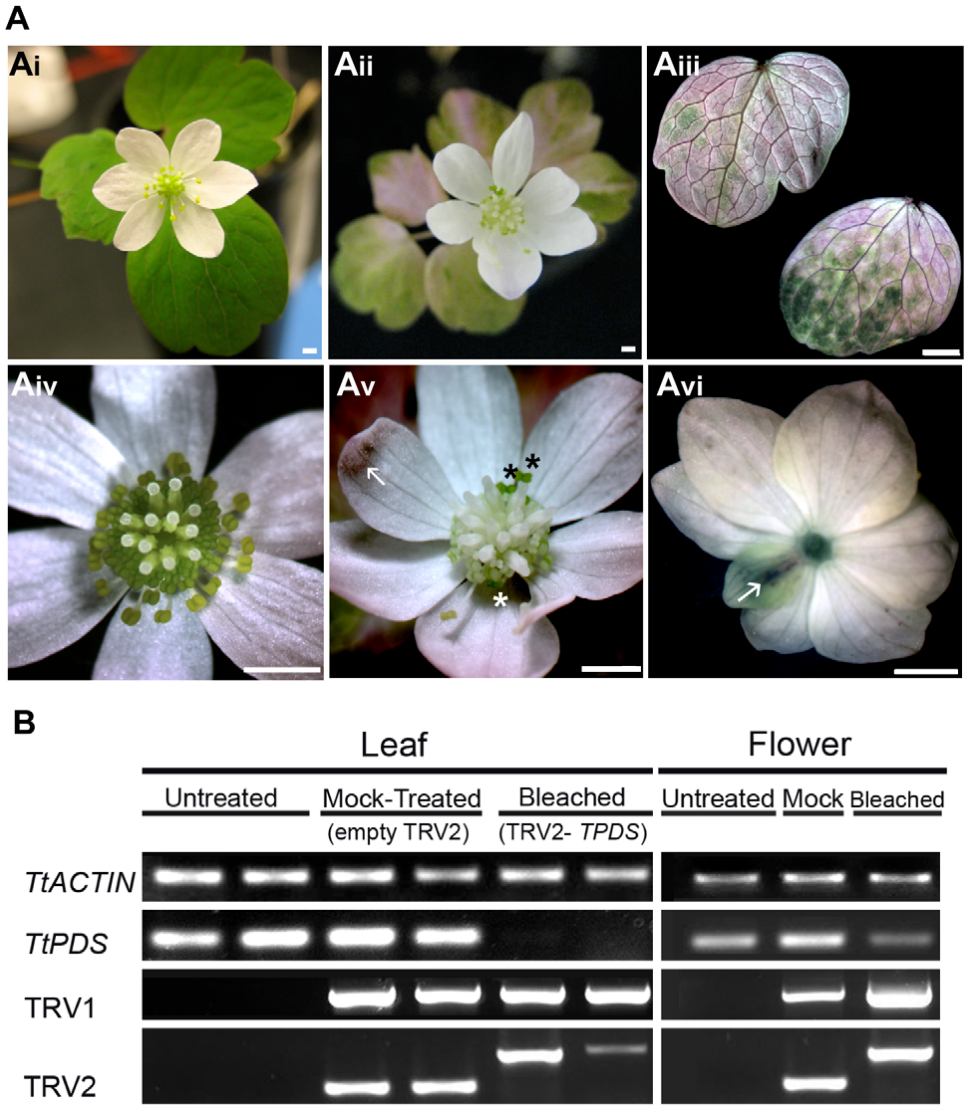
Virus-induced gene silencing of *TtPDS* causes photobleaching in leaves and flowers of *Thalictrum thalictroides*. **A: Flower and leaf** ***TtPDS*** **silencing phenotypes compared to controls.** Ai: Untreated flower of *T. thalictroides*, note green leaflets and green/yellow floral center; Aii: TRV2-*TPDS* treated plant, showing partial photobleaching of leaflets that appear variegated; Aiii: Detail of varying degrees of photobleaching in leaflets; Aiv: Detail of untreated flower, note that carpels and young stamens are normally photosynthetic (green); Av: Detail of treated flower showing silencing in stamens and carpels, three older stamens are not photobleached and therefore look green (asterisks), a patch of necrotic tissue (a viral effect) is indicated with an arrow; Avi: empty TRV2 mock-control flower showing background viral effects: arrow points to reduced sepal with patch of necrotic tissue. Scale bar  = 1 mm. **B: Comparative expression of** ***TtPDS*** **in leaves and flowers of** ***T. thalictroides*** **plants treated with TRV2-** ***TPDS*** **, relative to controls.** Untreated and mock-treated (empty TRV2) plants are compared to TRV2-*TPDS* treated plants showing photobleached leaves (left panels) or flowers (right panels). Reverse-transcriptase PCR was performed with locus-specific primers to the housekeeping gene *TtACTIN* (loading control), to endogenous *TtPDS* and to the viral transcripts TRV1/TRV2. For TRV2: larger band results from the presence of insert, smaller band from an empty TRV2 (mock control).

In order to achieve floral silencing, we infiltrated dormant, bare-root plants of the spring ephemeral hermaphrodite *T. thalictroides* (Fig. 3Ai). In this species, flowers develop from a fleshy root (a small tuber) simultaneously with leaves in the second year. Therefore, photobleaching due to *PDS* silencing can be rapidly detected (less than 2 weeks, and as little as 4 days) not only in leaves (Fig. 3Aii, Aiii detail), but also in photosynthetic carpels and young stamens (compare Fig. 3Aiv-Av).

Survival in this experiment was only 25% (5 out of 20 treated plants), presumably due to the plants being young; the small tender tubers did not respond well to wounding and longer infiltration time. Age at infiltration was especially critical for bleached plants; in the absence of green photosynthetic leaves, the young tubers did not have enough stored metabolites to sustain them and the plants died. Only 2 bleached plants survived, and one flowered. Subsequently, we have experimented with older plants, with significantly increased survival rates. All mock-treated plants survived, and approximately two thirds flowered (10/15); of these, most (8/10) showed varying degrees of necrosis (black spots) and reduced sepal size (Fig. 3Avi). These phenotypes were interpreted as background viral effect, and discounted from further analyses of floral silencing.

Detection of TRV1 and TRV2 transcripts in cDNA provided evidence that silencing was due to the viral treatment (Fig. 3B). Downregulation of *TtPDS* was most marked in photobleached leaves, where expression was not even detectable by RT-PCR (Fig. 3B left panels). *TtPDS* downregulation was less pronounced in flowers, where the bulk of the tissue (petaloid sepals) is white (Fig. 3B, right panels).

#### Silencing of an ortholog of the floral MADS box gene AGAMOUS in *Thalictrum clavatum*

**Figure 4:**
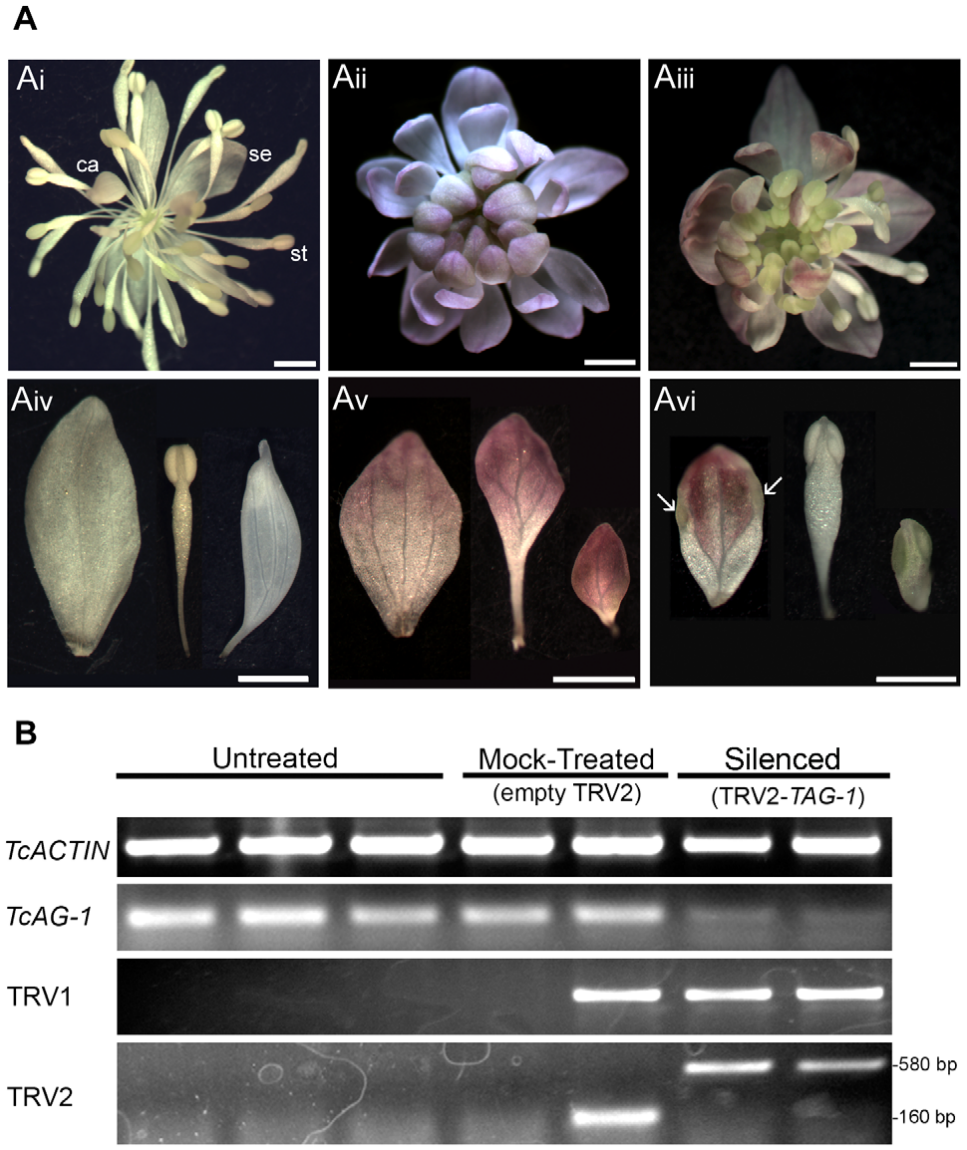
Virus-Induced Gene Silencing of *Thalictrum clavatum AGAMOUS* ortholog *TcAG-1* results in homeotic floral phenotypes. **A: Flower silencing phenotypes of** ***TcAG-1*** **, relative to controls.** Ai, Untreated flower of *T. clavatum* showing sepals (se), stamens (st) and carpels (ca); Aii: strongly silenced flower in TRV2-*TAG-1* treated plant, showing an array of sepals and no stamens nor carpels, all reproductive organs have been homeotically converted to sepals; Aiii: intermediate phenotype with partial conversion of organs and some normal ones; Aiv: detail of dissected organs in an untreated flower (sepal, stamen, carpel, from left to right); Av: detail of all sepaloid dissected organs from a strong *TcAG-1* silencing phenotype (from the outside to the inside of the flower, left to right); Avi: detail of sample chimeric organs, arrows point to anther tissue on the edges of an internal “sepal”. Scale bar = 1 mm. **B: Gene expression by Reverse Transcriptase (RT)-PCR in** ***TcAG-1*** **silenced plants compared to controls.** Untreated and mock-treated (empty TRV2) plants are compared to TRV2-*TAG-1* treated plants showing strong homeotic conversions (Aii, Av). RT-PCR was performed with locus-specific primers to the housekeeping gene *ACTIN* (loading control); to the MADS box gene *TcAG-1* and to the viral transcripts TRV1/TRV2. For TRV2: larger bands result from the presence of insert, the smaller band from an empty TRV2 (mock control).

*T. clavatum* is a close relative of *T thalictroides* representing a different type of flower morphology, with smaller pink/white petaloid sepals that fall off in mature flowers and prominent stamens with flattened, petaloid filaments (compare Figs. 3Ai and 4Ai). This species was treated with a TRV2-*ThtAG-1* single construct, to silence the ortholog of the *Arabidopsis* floral MADS box gene *AGAMOUS*, described earlier [30]. Silenced flowers showed homeotic conversion of stamens and carpels to petaloid sepals (Fig 4A, the entire genus *Thalictrum* lacks petals), as described for *ag* loss of function mutants in *Arabidopsis* [32]. Untreated flowers consist of 4–6 white sepals, 26–39 stamens with flattened petaloid filaments and 5–9 stalked carpels (flower counts based on 15 flowers from 5 plants) (Fig. 4Ai, Aiv). No viral effects were detected in the TRV2 empty controls for this species. Two of the treated plants showed strong phenotypes (Fig. 4Aii) in 9 and 15 flowers respectively, consisting of complete conversion of reproductive organs (stamens and carpels) into sterile organs (sepals) of different size and shape (different degrees of narrowing at the base); no effects were evident in sepals (Fig. 4Av). Intermediate phenotypes were also observed in 3–4 flowers per plant (Fig. 4Aiii), consisting of partially converted organs, including sepaloid organs with anther tissue (Fig. 4Avi, arrows) and staminoid organs with unusually expanded filaments, becoming reduced in size towards the center of the flower (Fig. 4Avi). While intermediate organs with staminoid features were common, none of the silenced flowers had carpels. Silenced flowers had immature organs that continued to develop in the center throughout the life of the flower; consistent with the role of *AG* in flower determinacy in *Arabidopsis* [32].

Phenotypes were validated at the molecular level: all untreated and mock-treated plants tested had higher expression of *TcAG-1* than treated plants, as shown by RT-PCR on individual flowers, relative to *ACTIN* (Fig. 4B). TRV transcripts were present in treated-silenced and one of the two mock treated flowers shown (like in the other species, infiltration efficiency is not 100%) and absent from untreated flowers, as expected (Fig. 4B). Larger bands in TRV2 (580 bp) correspond to the presence of the *TAG-1* insert in treated plants, whereas smaller bands (160 bp) correspond to an empty TRV2 in the mock controls (as explained for Figs. 2A and 3B; all inserts are approximately 400 bp).

## Discussion

*Thalictrum* is one of the most species-rich genera in the family Ranunculaceae and has a key phylogenetic place at the base of the eudicots, which represent a smaller radiation nested within the major angiosperm radiation [33]. This basal position, combined with the retention of ancestral floral features, provides a window into past scenarios of flower evolution. It is this particular combination of key phylogenetic position and floral diversity that makes *Thalictrum* a promising model plant lineage for evo-devo studies [34].

Recently, VIGS has been employed in a variety of plant systems as a reverse genetics approach [35]. It is becoming a powerful tool in the area of evolution of plant development, allowing for functional studies of floral transcription factors across the angiosperm phylogeny, including early-diverging eudicots [27], [36]. Our demonstration that VIGS can be implemented efficiently to silence a carotenoid pathway gene, as well as a floral transcription factor in three species of *Thalictrum*, provides proof of the value of this type of approach in evolutionary studies involving early-diverging eudicots.

The successful implementation of VIGS in leaves and flowers of *Thalictrum* species is a major step towards investigating gene function in this emerging model plant genus. Its amenability to vacuum infiltration of seedlings or dormant plants underscores the versatility of these herbaceous perennials. Post-treatment survival rates for *T. dioicum* seedlings were amongst the highest observed for this infiltration method (97%), comparable to those reported previously in *Papaver* [24] and higher than those in the closely related *Aquilegia* [22]. Further, we observed a higher percentage of the plants showing photobleaching at 42% compared with 12% and 23% in the above studies.

Implementation of VIGS in *Thalictrum* broadens the already wide host range of tobacco rattle virus and further supports the use of VIGS in other, lesser known plant systems for which stable transgenic techniques are not yet available.

Moreover, *T. dioicum* is the only dioecious species emerging so far as a model system among basal eudicots [34]. Comparative functional analyses within this genus, amongst hermaphroditic (*T. thalictroides* and *T. clavatum*) and dioecious species (*T. dioicum*), will facilitate studies of the genetic basis for the evolution of sexual dimorphism.

Most importantly, the use of VIGS has allowed us to carry out functional analyses within *Thalictrum* rather than relying on transformation into established model systems, with its inherent limitation to biochemically rather than physiologically informative results. A heterologous approach also deters the investigation of subtle functional differences amongst duplicated genes present in *Thalictrum* and widespread in the Ranunculaceae [30], [31], due to the lack of a suitable molecular environment. The above limitations are widespread and would ultimately prevent a thorough investigation of the origin and evolution of key regulators of development that may have shaped the evolution of angiosperms using different pathways such as sub or neo-functionalization [37].

Certain species of *Thalictrum* are economically significant in the pharmacological [4] and horticultural industries [38]. The development of this technique will facilitate the study of gene function of clinically relevant secondary metabolite biosynthesis in *Thalictrum*. Many species of *Thalictrum*, including the two hermaphrodites in this study, are sold as ornamentals. This study enables the exploration of the genetic basis of existing varieties and the creation of new, showier ones (such as the “double” flowers resulting from *AG* silencing, Fig 4Aii), a desirable goal for the floriculture industry.

In conclusion, we have shown that VIGS is an effective tool to assess gene function in three species of *Thalictrum*, resulting in leaf and floral phenotypes. Silencing of the floral MADS box gene *TAG-1* caused homeotic conversions of stamens and carpels into sepals, as predicted by the ABC model [32]; silencing of *TPDS* produced the expected photobleached phenotype in leaves and flowers. The *Thalictrum* ortholog of *PDS* is a useful vegetative marker to quickly identify plants that are undergoing silencing, mainly in green leaves and additionally in species with green flowers (most of the wind-pollinated taxa), or green floral parts during early development (*T. thalictroides* and *T. clavatum*). Photobleaching can, however, be detrimental to plant growth and survival, especially in young plants. Therefore, the use of a marker gene in double constructs must be considered carefully, and may not be justified in cases where there is an expectation for a well-defined phenotype. With these caveats, high survival rates in seedlings and potentially improved ones on older tubers, combined with high infiltration efficiency and silencing rates, make VIGS promising for functional studies in these and related species.

With the prospect of a full-length transcriptome for *T. thalictroides* through the 1KP project (Univ. of Alberta, Canada), the ability to test genes or whole gene families by VIGS in this genus is especially timely [9]. In order to build a toolbox for an emerging model system, it is indispensable to have a mechanism to assess gene function [10]. Here, we have successfully adapted a tool for functional studies, which is rapid, relatively simple to implement and shows high promise for a comparative functional approach in *Thalictrum* and beyond.

## Materials and Methods

### Plant Materials

*Thalictrum dioicum* seeds (greenhouse-collected from wild accessions) were imbibed in distilled water for 2 days at 4°C, then sown on Turface soil medium (Buffalo Grove, IL) 288-cell trays or in Oasis Wedge system foam medium (Kent, OH) 102-cell trays. Trays with sown seed were stratified for six weeks at 4°C covered in plastic to avoid evaporation, then uncovered and transferred to the UW greenhouse (20°C, 14–16 hr days), where germination was seen within approximately 2 weeks. Seedlings with 2–3 true leaves were used for further experiments. Flowering of *T. dioicum* seedlings typically occurred 6 months after sowing.

*T. thalictroides* bare root plants were purchased from nurseries and kept at 4°C in peat moss until infiltration.

*T. clavatum* plants that had died back were vernalized in a 4°C room for 8 weeks, the small tubers were then dug up and used in the experiments.

Voucher specimens for the three species in this study are: *T. dioicum*, V. Di Stilio 101 (A); *T. thalictroides* V. Di Stilio 124 (WTU) and *T. clavatum*, V. Di Stilio 127 (WTU).

### Cloning of Thalictrum PDS

In order to clone the PDS ortholog, total RNA was isolated from *Thalictrum dioicum* and *T. thalictroides* leaves using TRIzol® Reagent (Invitrogen, Carlsbad, CA), following manufacturer's instructions. Samples were treated with amplification-grade DNaseI (Invitrogen, Carlsbad, CA), followed by First-Strand Synthesis with Oligo (dT) using the SuperScript III® System (Invitrogen, Carlsbad, CA). A 441bp fragment of the *Thalictrum dioicum* ortholog of *PDS (TdPDS)* was amplified by PCR using PDS-F2-XbaI and PDS-R3-BamH1 primers [22] and cloned into pCR2.1 using the TA cloning kit (Invitrogen, Carlsbad, CA). Three positive clones were verified by sequencing (Biochemistry DNA Sequencing Facility, University of Washington) and BLAST search (NCBI). In order to design endogenous *TdPDS* specific primers, we cloned a longer fragment of *TdPDS*. To that end we used primers designed to *Aquilegia vulgaris PDS* (GenBank DQ923721, (22)): AqPDS specific F1 5′-AAT GCC AAG CAA GCC AGG AG -3′ and AqPDS specific R1 5′-TCA GGG AAG AGT TTC GCA AGC -3′, at 53°C and 30 cycles. The resulting 830 bp partial coding sequence (*TdPDS*, deposited as GenBank FJ457899) was used to design primers outside of the region contained in the silencing construct.

The same approach was applied to isolate the orthologous *PDS* fragment from *T. thalictroides* (*TtPDS*, deposited as GenBank HM48111), which was similarly used to design RT-PCR locus-specific primers outside of the region used in the silencing construct.

### Preparation of the TRV2-*TdPDS* construct

The *TdPDS* clone was PCR amplified using the forward and reverse primers described above with added restriction sites for cloning: 5′-AGTGGATCCCAGCCGATTTGATTTCCCAGAT-3′ (TdPDS_F_BamHI) and 5′-AAGCTCGAGGAGAATTGAGTGGGACTTCACCA-3′ (TdPD*S*_R_XhoI). The resulting amplicon was gel purified using QIAquick Gel Extraction Kit (Qiagen, Valencia, CA). Dr. Dinesh Kumar kindly authorized us to use the TRV1 and TRV2 vector system developed in his laboratory. The TRV2 plasmid and *TdPDS* fragment were digested with BamH1 and Xho1 (New England Biolabs, Ipswich, MA), ligated using T4 DNA ligase (Invitrogen, Carlsbad, CA) and transformed into One Shot® TOP10 Chemically Competent *E. coli* (Invitrogen, Carlsbad, CA). Colonies were selected on LB plates containing 50 µg/ml of Kanamycin and the presence of insert was confirmed by PCR with primers spanning the Multiple Cloning Site of pTRV2 (156 F: 5′- TTA CTC AAG GAA GCA CGA TGA GC -3′ and 156 R: 5′- GAA CCG TAG TTT AAT GTC TTC GGG -3′) [22]. In the absence of insert, the expected size of the PCR product is 160 bp; in the presence of *TdPDS*, the resulting amplicon size should be 585 bp. TRV2-*TdPDS* plasmid was purified from a single positive colony using FastPlasmid Mini kit (Eppendorf, Hauppauge, NY), then confirmed by sequencing.

### Preparation of *Thalictrum AG-1* construct

Since the *TAG-1* locus is highly conserved within *Thalictrum* and even among genera of the Ranunculaceae [34], we used a *T. thalictroides* existing construct (TRV2-*TtAG-1*) on *T. clavatum*, after checking for sufficient homology between the two to elicit silencing. The complete coding region of *TcAG-1* was cloned (deposited as GenBank HM488113). Since both species share 99% nucleotide identity in the region used for silencing, we will refer to this construct as TRV2-*TAG-*1 (for *Thalictrum AG-1*). To prepare the silencing construct, flower bud cDNA of *T. thalictroides* was used as template in PCR with *AG-1* specific primers and added XbaI and BamHI restriction sites: TthAG1_fwd_xba1 (5′ AGG TCT AGA GCA ATG ATC GCT GCA AAC GAG 3′) and TthAG1_rev_BamHI (5′ AAT GGA TCC CAG ACA AAA TGC CAA GTC CCT C 3′). A PCR product of approximately 500 bp was excised from the agarose gel, and extracted using QiaQuick gel extraction kit (Qiagen, Valencia, CA). The resulting DNA was digested with XbaI/BamHI restriction enzymes (New England Biolabs, Ipswich, MA) to create sticky ends and ligated into a similarly digested TRV2 vector, yielding the TRV2*-TtAG-1* construct. The identity of the insert was confirmed by sequencing.

### Transformation of Agrobacteria with TRV constructs

Electrocompetent Agrobacteria GV3101 were prepared as described elsewhere [39] and transformed with 2 µl of pTRV2-*TdPDS*, pTRV2-*TAG-*1, pTRV2 (empty) or pTRV1. Electroporation was carried out at 2.4 Kv for 5 ms on a MicroPulser Electroporator (Bio-Rad Laboratories, Hercules, CA). Cells were selected on LB plates containing 50 µg/ml Kanamycin, 25 µg/ml Rifampicin and 50 µg/ml Gentamycin. Colonies were confirmed by PCR as explained above, sequenced and stored as glycerol stocks at −80°C.

### Infiltration of *T. dioicum* seedlings

In order to achieve suppression of expression of *TdPDS*, a total of 117 *T. dioicum* seedlings at the 2–3-leaf stage across 3 independent experiments were infiltrated with *Agrobacterium* containing pTRV1 and pTRV2-*TdPDS*. A negative control (or mock treatment) consisted of infiltrating 50 seedlings with a mixture of pTRV1 and empty pTRV2 to test for background viral effects; another group of 5 seedlings was left untreated and grown under the same conditions.

*Agrobacteria* were prepared for infiltration following [22], with modifications. Starter overnight LB cultures (5 ml) of pTRV1, pTRV2-*TdPDS* and empty pTRV2 were grown overnight with selective antibiotics and used subsequently to inoculate 50 ml and 500 ml cultures. 1 M MES (2-(4-Morpholino)-Ethane Sulfonic Acid) and 0.1 M Acetosyringone (3′,5′ -Dimethoxy-4′-hydroxyacetophenone) were added to the final cultures. These were grown to an OD_600_ of 2.0, then centrifuged at 4,000 g for 15 min at 4°C. Cells were resuspended in infiltration medium (10 mM MES, 20 µM acetosyringone, and 10 mM MgCl_2_) to a final OD_600_ of 2.0 and incubated for 3 hrs at room temperature. Cultures of pTRV1 were mixed in a 1∶1 ratio in a 2-liter plastic container with either pTRV2-*TdPDS* (silencing treatment) or empty TRV2 cultures (mock control), adding 100 ul/l Silwet L-77 (Lehle Seeds, Round Rock, TX) as a surfactant. Seedlings were removed from Turface or foam medium, roots were rinsed in distilled water and whole seedlings were submerged in infiltration medium containing either pTRV1 mixed with pTRV2- *TdPDS* or TRV1 mixed with empty TRV2 (mock control). A −100 kPa vacuum was applied in a chamber for 2 minutes. Following infiltration, seedlings were potted in soil and grown in the greenhouse. Photobleaching of leaves, detectable two weeks after infiltration, was scored for up to 4 months following inoculation. Photobleached, variegated and green leaves were collected starting at 3 weeks post infiltration, flash-frozen in liquid nitrogen and stored at −80°C until processing.

In order to record photobleached phenotypes, plants were photographed using a hand held digital camera and a dissecting microscope (Nikon SMZ800, Nikon Instruments Inc., Melville, NY) equipped with a QImaging MicroPublisher 3.3 RTV digital camera (Surrey, BC, Canada). Images were processed in Adobe® Photoshop® CS2 v 9.0.2 and figures were assembled using Adobe® Illustrator®CS2 v. 12.0.1.

### Infiltration of *T. thalictroides* and *T. clavatum* dormant plants

Dormant underground tubers of *T. thalictroides* and *T. clavatum* were cleaned of soil, then kept in the dark covered in wet paper towels until infiltration media were ready. The small tubers were wounded lightly before infiltration using a clean razor blade to facilitate the entrance of *Agrobacteria* carrying the TRV plasmids. Vacuum infiltration was carried out as above, except the infiltration time was longer: 10 min for *T. thalictroides* and 5 minutes for *T. clavatum* (smaller tubers).

Given the high conservation of the *PDS* locus, silencing constructs can be used successfully across species. Therefore, *T. thalictroides* plants were treated with the available *T. dioicum PDS* construct, TRV2-*TdPDS*, which is 99% identical at the nucleotide level over the silencing fragment. Similarly, *T. clavatum* was treated with a *T. thalictroides AG-1* construct (99% identical, see details above). For simplicity, these constructs are referred to as TRV2-*TPDS* and TRV2-*TAG-1* throughout the text. Mock-treated controls were infiltrated identically, except the TRV2 vector did not contain an insert. Untreated plants were given identical treatment, but without infiltration.

After infiltration, tubers where potted in 2.5” Deepots™ (Stuewe & sons, Tangent, OR) using Sunshine Mix #4 soil (Sun Gro, Bellevue, WA) without watering and transferred to the UW greenhouses (20°C, 14–16 hrs light), where they flowered in less than 2 weeks (*T. thalictroides*) to 3 weeks (*T. clavatum*). Pots were covered with plastic for 24 hours, then uncovered and watered twice a week for the duration of flowering.

Plants were monitored daily throughout the flowering period. Once flowers started to show homeotically converted organs, they were collected and flash frozen in liquid nitrogen for later analysis. Flowers from mock-treated and untreated plants were collected similarly to use as controls.

### Semi-quantitative analyses by RT-PCR

Total RNA was extracted from frozen leaves as described above. First strand synthesis was carried out using the pTRV1 specific primer OYL 198 (5′- GTA AAA TCA TTG ATA ACA ACA CAG ACA AAC -3′) [24], pTRV2 specific primer 156 R [22], or Oligo (dT). A set of reactions without Reverse Transcriptase was used to control for presence of genomic DNA. Reverse transcriptase (RT)-PCR was performed for 25 cycles using pTRV1 specific primers OYL195 (5′- CTT GAA GAA GAA GAC TTT CGA AGT CTC -3′) and OYL198 [24], 51°C anneal; TRV2 specific primers 156 F and 156 R [22], 51°C anneal; and *Thalictrum ACTIN* specific primers TthActin for 2 (5′-GCAGAACGGGAAATTGTCCGC-3′ and TthActin rev 2 (5′- CCTGCAGCTTCCATTCCGATCA-3′), 58°C anneal; or endogenous *TdPDS* specific primers TdPDS_F_RT (5′-TGA ATA ATG ATG GAA CCG TG-3′) and TdPDS_R_RT (5′-GTC AGC ATA CAC ACT CAA AAG G-3′), 50°C anneal.

RT-PCR products were run on a 1.2% agarose gel. For the *T. dioicum* experiment, *TdPDS* band intensity was quantified using ImageJ (NIH), normalized against *TdACTIN* controls. The statistical significance of the difference in normalized *TdPDS* expression among treatments was tested by one-tailed ANOVA followed by Tuckey test in JMP (statistical discovery software, Cary, NC).

Untreated, mock treated and photobleached leaf and floral tissue of *T. thalictroides* was collected, processed for cDNA and assessed for gene expression as explained above, except the forward *PDS* primer used to detect expression in cDNA was adjusted to be species-specific: *TthPDS*_F_RT (5′- TGA ACA ACG ATG GAA CCG TG-3′), and 32 cycles (at 53°C) were run on floral tissue due to lower levels of PDS compared to leaves.

Silenced flowers of TRV2-*TthAG-1* treated *T. clavatum* plants that showed homeotic conversions were similarly collected, processed and compared to controls (untreated and empty TRV2). *Thalictrum AG-1* specific primers TthAG1_fwd_qPCR (5′-AGTCTCTCAGCAATCTCAATATCAGGG-3′) and TthAG1_rev_qPCR (5′-GCCCTGAGATACTTGTTATCAGRTCTGC-3′) for 23 cycles at 53°C, were used to determine *TcAG-1* expression levels. Previously designed *PDS* and *ACTIN* primers for *T. thalictroides* were used on *T.clavatum*, due to high sequence similarity between the two closely related species.
